# Characterization and Anti-Aging Potency of Phenolic Compounds in Xianhu Tea Extracts

**DOI:** 10.3390/foods14050737

**Published:** 2025-02-21

**Authors:** Guangwen Zhang, Wenwen Jiang, Qing Hu, Jianming Luo, Xichun Peng

**Affiliations:** Department of Food Science and Engineering, Jinan University, Guangzhou 510632, China; tzgw@jnu.edu.cn (G.Z.); 13070222797@163.com (W.J.); dip9786@126.com (Q.H.); luojm@jnu.edu.cn (J.L.)

**Keywords:** anti-aging, phenols, antioxidation, *C. elegans*

## Abstract

The health benefits of tea are primarily attributed to its chemical composition, particularly phenolic compounds. As a renowned tea from Guangdong, China, Xianhu tea (XHT) has not been thoroughly studied in terms of its phenolic composition or health-promoting properties. This study characterized the phenolic compounds in Xianhu tea water extract (XHT) using liquid chromatography–tandem mass spectrometry (LC-MS/MS) and evaluated its antioxidant activity in vitro. Furthermore, the effects of XHT extracts on reactive oxygen species (ROS), malondialdehyde (MDA), superoxide dismutase (SOD), catalase (CAT), lipofuscin levels, and lifespan in *Caenorhabditis elegans* were assessed, alongside their modulation of aging-related genes. Compared with Xinyang Maojian tea water extract (XYMJ) and Yingde black tea water extract (YDBT), XHT exhibited a significantly higher polyphenol content, with 23 phenolic compounds identified as characteristic markers. XHT demonstrated superior 2,2-diphenyl-1-picrylhydrazyl (DPPH) radical-scavenging and 2,2′-azinobis-(3-ethylbenzthiazoline-6-sulfonic acid) (ABTS) inhibition capacities, the greatest reductions in ROS, MDA, and lipofuscin levels, and the highest upregulation of SOD and CAT activities. The lifespan was 23.50% and 21.07% significantly longer than nematodes in the control group. Moreover, XHT modulated 13 aging-related genes, which strongly correlated with the 23 identified phenolic compounds. The research results of the above indicators were all obtained without affecting the normal feeding and reproductive capabilities of *C. elegans*. These findings suggest that these phenolics are the key bioactive components responsible for the anti-aging effects of XHT.

## 1. Introduction

Tea has been shown to confer numerous health benefits, including antioxidant, anti-aging, immunomodulatory, anticancer, cardiovascular protective, antidiabetic, anti-obesity, and hepatoprotective effects [1]. These effects are closely linked to tea’s active chemical composition, particularly its polyphenols, including flavonoids and phenolic acids [2,3]. Variations in cultivation methods, processing techniques, tea varieties, and growing environments influence tea’s polyphenol content and bioactivity [4,5,6]. Currently, numerous well-known teas available on the market have been extensively studied, demonstrating to possess antioxidant and anti-aging effects. However, Xianhu tea, a renowned tea variety from the Guangdong province, has not been subjected to similar research. As a result, there is a significant gap in our understanding of its antioxidant and anti-aging properties, as well as any unique characteristics it may possess. Therefore, researching the anti-aging effects of Xianhu tea is of paramount importance. Xianhu tea is processed from the tea bushes (*Theaceae* Mirb.) located in Heyuan city, Guangdong province. The region’s high altitude (600–800 m), abundant sunshine, rainfall, and fertile acidic red soil create optimal conditions for tea growth. In addition, moderate planting density, proper ventilation, light management, natural fertilizers, and specialized processing methods, including quick-kill and low-temperature drying techniques, contribute to XHT’s superior quality and unique flavor [7,8]. These factors collectively suggest that Xianhu tea may exhibit unique polyphenol composition, as well as distinct biological activities compared to other teas.

Aging and longevity are closely associated with oxidative stress [9]. Aging is a universal physiological process, accompanied by systemic alterations in cellular structure resulting from modifications in metabolic and signal transduction pathways [10], including the autophagy-related target of rapamycin (TOR) signaling pathway, the insulin/insulin-like growth factor 1 (IGF-1) signaling (IIS) pathway, the mitochondrial-related functional signaling pathway, and the adenosine monophosphate-activated protein kinase (AMPK) signaling pathway [10,11]. To mitigate the impacts of oxidative stress caused by aging, organisms activate intercellular antioxidant enzyme systems, including superoxide dismutase (SOD), catalase (CAT), and glutathione peroxidase [12]. Nevertheless, as the endogenous antioxidant system diminishes with age, the administration of exogenous antioxidants to stimulate this system represents a crucial strategy for decelerating the aging process and enhancing health [12]. Polyphenols, especially tea polyphenols, have been demonstrated to have potent antioxidant effects that mitigate age-related chronic diseases [13].

*Caenorhabditis elegans* (*C. elegans*), with over 3000 mutant strains available, has been employed extensively in studies investigating oxidative stress, aging, and longevity [14,15]. As this nematode and humans share oxidative stress, lifespan regulation, and aging disease signaling pathways, *C. elegans* was considered a reliable in vivo model for the high-throughput screening and validation of natural antioxidants with anti-aging activity [16].

As mentioned, Xianhu tea ought to have some specific phenolic content and beneficial effects due to differences in its growth environment and processing technology from other teas. However, its phenolic contents and types and health-beneficial effects have not been previously reported. In this study, Yingde black tea and Xinyang Maojian, which are distinguished by their remarkable antioxidant properties and significant market presence, were chosen as reference samples for comparative analysis with Xianhu tea. Such a comparison serves to accentuate the distinctive antioxidant attributes of Xianhu tea and elucidate its latent market potential. Xianhu tea, a well-known tea variety in the Guangdong province, has been relatively under-researched despite its significant production volume and high-quality products. Although Xianhu tea is produced in large quantities and is of exceptional quality, its market share remains small, and it has not been fully utilized. Conducting in-depth research on Xianhu tea will not only enrich the available data on this tea variety but also provide a scientific basis for incorporating Xianhu tea and its active components into antioxidant and anti-aging products. Additionally, such research will lay the foundation for the development of Xianhu tea-related products and the expansion of its market share. Our study aims to analyze the composition of Xianhu tea, in particular the types and amounts of phenolics, figure out its health effects, including its antioxidative effect, anti-aging efficacy, and lifespan extension, using Xinyang Maojian tea and Yingde black tea for comparison, and finally identify the phenolic constituents that are most possibly related to the tea’s properties. Therefore, conducting the aforementioned research will effectively enrich data resources on Xianhu tea, demonstrating its significant academic research value and the necessity of the study.

## 2. Materials and Methods

### 2.1. Chemicals and Reagents

Folin–Ciocalteu’s phenol reagent was employed (Shanghai Macklin Biochemical Technology Co., Ltd., Shanghai, China), alongside superoxide dismutase (SOD), catalase (CAT), malondialdehyde (MDA), and bicinchoninic acid (BCA) detection kits (Biyuntian Biotechnology Co., Ltd., Shanghai, China). In addition, 2′,7′-dichlorodihydrofluorescein diacetate (DCFH-DA, 98%) was used (Sigma-Aldrich Trading Co., Ltd., Shanghai, China), along with ChamQ Universal SYBR qPCR Master Mix and HiScript IV All-in-One Ultra RT SuperMix for qPCR (Nanjing Vazyme Biotech Co., Ltd., Nanjing, China). Finally, 5-fluorouracil (5-FUDR) was employed (Shanghai Acmec Biochemical Technology Co., Ltd., Shanghai, China).

Xianhu tea was collected from the tea industry planting base of Xianhushan Agricultural Development Co., Ltd., Heyuan city, Guangdong province, China, while Xinyang Maojian tea was collected from Xinyang city, Henan province, China, and Yingde black tea was from Yingde city, Guangdong province, China.

### 2.2. Sample Preparation

Xianhu tea (XHT) was harvested in Heyan city, Guangdong province, in mid-April 2023. Tea leaves underwent stir-frying at 270–280 °C, kneading for 30–40 min, and infrared drying at 90–100 °C. Comparative samples of Yingde black tea (YDBT) and Xinyang Maojian tea (XYMJ) were purchased from local markets. All tea samples were ground into a powder, and 60g of powder was extracted with 1200 mL of ultrapure water (1:20 *w*/*v*) at 100 °C for 2 h under micro-boiling conditions. The extract was filtered, re-extracted, and combined. The resulting solution was centrifuged at 3000 rpm for 15 min using a centrifuge of the brand Gaco, model TD5A, and the supernatant was concentrated to one-third of its original volume. Finally, the concentrate was freeze-dried for 48 h to obtain tea powder. After 48 h of freeze-drying, the moisture content of the resulting freeze-dried powder was approximately 6%, and the powder was in a non-adhesive state. The resulting powder was sieved through a 400-mesh screen for the subsequent experiments.

### 2.3. Determination of Total Phenols, Polysaccharides, and Flavonoids

Total phenolic content was measured using the Folin–Ciocalteu reagent with gallic acid as the standard [17]. Based on this method, some minor improvements could be made. We transferred 1.0 mL of gallic acid working solution into a 10 mL centrifuge tube, added 2.5 mL of Folin–Ciocalteu reagent, and mixed well. Then, we added 2.5 mL of 15% Na_2_CO_3_ solution and diluted to the mark with water to a total volume of 10 mL, mixing thoroughly. We incubated the mixture in a water bath at 40 °C for 60 min, followed by cooling at room temperature for 20 min. We measured the absorbance at 750 nm. We then plotted the standard curve with concentration as the *x*-axis and absorbance as the *y*-axis. The resulting standard curve equation was *y* = 0.0034*x* + 0.0583, with R^2^ = 0.9928. Polysaccharides were quantified using the phenol–sulfuric acid method [18]. We took 1.0 mL of standard solution and 1.0 mL of sample solution separately into test tubes, added 1.0 mL of 5% phenol solution, mixed well, quickly added 5.0 mL of concentrated sulfuric acid, mixed again, heated in a boiling water bath for 15 min, cooled to room temperature, and measured the absorbance at 490 nm. The equation of the standard curve obtained was *y* = 4.8457*x* + 0.07, with R^2^ = 0.9993. The flavonoid content was determined via AlCl_3_ colorimetry [19]. We placed different volumes of rutin reference solution into 10 mL stoppered colorimetric tubes. Then, we sequentially added 2 mL of acetate buffer solution at pH 5.4 and 1 mL of 2% AlCl_3_ solution. We diluted to the mark with 70% ethanol and mixed thoroughly. We allowed the mixture to stand at 30 °C for 12 min. We used the solution without rutin as the blank control. We measured the absorbance at 285 nm. We performed linear regression of the rutin solution concentration C (mg/mL) against the absorbance value A to plot the standard curve. The resulting standard curve equation was *y* = 1.7391*x* + 0.065, with R^2^ = 0.9959.

### 2.4. LC-MS/MS Analysis of Phenolic Compounds

Sample preparation was slightly modified based on previous studies [20,21]. Fifty micrograms of (50 mg) lyophilized sample was added into 600 μL of water–methanol (*v/v* = 1:2, containing internal standard succinic acid-2,2,3,3-d4 and salicylic acid-d4) solution, and 400 μL of chloroform was then added. The mixture containing the sample was ground with a grinder at 60 Hz for 2 min and ultrasonically extracted in an ice-water bath for 20 min. The extract was then centrifuged (13,000 rpm) at 4 °C for 10 min, and 200 μL of supernatant was then placed into a new EP tube. Another 400 μL of water–methanol (*v/v* = 1:2, containing the internal standard succinic acid-2,2,3,3-d4 and salicylic acid-d4) solution was added into the residue, vortexed for 1 min, and extracted by ultrasound for 20 min. The second extract was centrifuged (13,000 rpm) at 4 °C for 10 min, and 200 μL of supernatant was taken and combined with the previous 200 μL of supernatant for a total volume of 400 μL. The 400 μL of supernatant was evaporated to dryness, then re-dissolved in 200 μL of water–methanol solution (*v/v* = 18:7, containing the internal standard L-2-chlorophenylalanine). The re-dissolved solution was vortexed for 30 s, followed by sonication for 2 min, and it was allowed to stand for 2 h at −20 °C. After that, the solution was centrifuged (13,000 rpm) at 4 °C for 10 min. Supernatant with a volume of 150 μL was transferred to a brown LC injection vial and stored at −80 °C until the online analysis. Quality control (QC) samples were prepared by mixing equal volumes of extracts from all the samples.

The types and amounts of phenolics were analyzed by liquid chromatography–tandem mass spectrometry (LC-MS/MS). The chromatographic conditions were as follows: column ACQUITY UPLC HSS T3 (100 × 2.1 mm, i.d., 1.8 μm; Waters, Milford, CT, USA) was used, 0.1% each of formic acid–water solution (A) and acetonitrile (B) was used as the mobile phase, the flow rate was set at 0.35 mL/min, the column temperature was set at 40 °C, and the sample size was 20 μL. The gradient elution program was set as follows: 0–0.8 min, 95% A/5% B (*v/v*); 0.8–3 min, 95%~75% A/5%~25% B (*v/v*); 3–12 min 75%~56.2% A/25%~43.8% B (*v/v*); 12–13 min, 56.2%~1% A/43.8%~99% B (*v/v*); 13–14.4 min, 1% A/99% B (*v/v*); 14.41–15 min 1%~95% A/99%~5% B (*v/v*); and 15 min, 95% A/5% B (*v/v*).

The samples were ionized by electrospray ionization, and the mass spectrum signals were acquired in positive and negative ion scanning modes, respectively. The parameter settings were as follows: source temperature 500 °C, curtain gas 35 psi, nebulizer gas (GS1) 60 psi, drying gas (GS2) 50 psi, entrance potential of −10 V in negative mode, and 10 V in positive mode. The ion capillary voltage was set to −4500 V in negative mode and 5500 V in positive mode.

### 2.5. Determination of the DPPH Radical-Scavenging and ABTS Radical-Inhibitory Activities of XHT, YDBT, and XYMJ

The in vitro antioxidant activity of the tea samples was assessed via 2,2′-azido-bis (3-ethylbenzothiazoline-6-sulfonic acid) (ABTS) inhibition rate and 2,2-diphenyl-1-trinitrohydrazine (DPPH) clearance using vitamin C (VC) as the positive control. The ABTS inhibition capacity was measured based on a previous report [22]. The ABTS radical cation (ABTS^•+^) stock solution was generated by combining equivalent volumes of 7 mM ABTS and 2.45 mM K_2_S_2_O_8_ solutions, with subsequent incubation under dark conditions at ambient temperature (25 °C) for 12–16 h. The ABTS stock solution was diluted with anhydrous ethanol to give an absorbance value of 0.7 ± 0.02 at 734 nm and used as the ABTS working solution. Samples with different concentrations of distilled water with a volume of 20 μL were mixed with 180 μL of ABTS working solution, respectively, and then left to stand for 10 min. Then, the absorbance was measured at 734 nm. DPPH clearance was measured based on a previous report [23]. The reaction mixture was prepared by combining equivalent volumes of 0.2 mM DPPH solution and the pre-prepared sample solutions, followed by vortexing. The mixture was subsequently incubated under dark conditions at ambient temperature (25 °C) for 30 min and then subjected to centrifugation at 1000× *g* for 10 min. The supernatant was collected, and the absorbance was read at 517 nm.

### 2.6. Cultivation and Synchronization of Caenorhabditis elegans

Wild-type *Caenorhabditis elegans* N_2_ and uracil-deficient *Escherichia coli* OP50 were generous gifts from Mr. Yang Jing at the Institute of Biotransformation at Jinan University, Guangzhou, China. N_2_ wild-type *C. elegans*, frozen at −80 °C, were revived and cultured on an NGM medium supplemented with an appropriate amount of *E. coli* OP50 solution. NGM plates with *C. elegans* were incubated in an incubator at a temperature of 20 °C and a humidity of 60%. Once most *C. elegans* had grown to the L4 stage, a synchronization treatment was performed on a clean bench. Firstly, nematodes were collected in tubes with 100 mL M9 buffer (containing 2 × 10^−5^ mM of KH_2_PO_4_, 4.5 × 10^−5^ mM of Na_2_HPO_4_·12H_2_O, 1.03 × 10^−4^ mM of NaCl, and 1 × 10^−6^ mM of MgSO_4_), centrifuged at 3000 rpm for 2 min, and then allowed to stand for 1 min. Next, the supernatant was discarded, and 1 mL of cracking liquid (containing 5 × 10^−4^ M of NaOH and 20% of NaClO, ready to use) was added to each tube to lyse for 4~5 min, centrifuged for 1 min, and then the supernatant was discarded. Finally, 1.5 mL of M9 buffer was added to each tube, centrifuged for 1 min, and the supernatant was discarded. This final step was repeated 3 times. Eggs of the nematodes were collected after the above treatments and cultured on NGM plates coated with *E. coli* OP50 solution. *C. elegans* at the L4 stage were obtained after incubation in an incubator with constant temperature and humidity for 56 h. These nematodes were then used to investigate the effects of XHT extracts on health with the YDBT extracts and XYMJ extracts as a comparison. For the tests using *C. elegans* as the animal models, the tea sample concentrations of 1 mg/mL and 4 mg/mL were selected to be the low (L) and high (H) concentrations, based on the findings in our pilot study that samples at these 2 concentrations did not affect the normal growth of nematodes.

### 2.7. Dietary Restriction

Four conical flasks (100 mL capacity), each containing 50 mL of LB medium, were autoclaved at 121 °C for 30 min. The *E. coli* OP50 monoclonal clone was incubated in the LB liquid medium with a volume of 50 mL at 37 °C for 12 h. Each sterile aqueous extract (XHT, YDBT, and XYMJ) was individually introduced into separate sterile conical flasks (100 μL extract per flask) containing 50 mL of LB broth medium. For the control group, an equal volume of sterile distilled water was added. Subsequently, 100 µL of the *E. coli* OP50 solution was introduced to each of the four bottles. The flasks were placed in an incubator set at 37 °C and cultured at 140 rpm for six hours. The OD600 was measured at one-hour intervals.

### 2.8. Determination of the Effect of XHT, YDBT, and XYMJ on Lifetime

One hundred microliters (100 μL) of *E. coli* OP50 solution was added to the plates (containing 5-fluorouracil) and air-dried. Sample solutions at varying concentrations (100 μL) were plated onto 60 mm NGM agar plates (containing 5-fluorouracil) seeded with *E. coli* OP50, while sterile distilled water (100 μL) was used as the control. Synchronized nematodes at the L4 stage were randomly picked and transferred to NGM plates containing 150 μM 5-fluorouracil. Seven groups, including XHT (L), XHT (H), YDBT (L), YDBT (H), XYMJ (L), XYMJ (H), and the control, were established, with 40 nematodes inoculated for each group. The day when the nematodes were transferred was recorded as day 0. The nematodes were then transferred to fresh plates every 3 days for the first 12 days, while they were transferred to fresh plates every 24 h for the remaining days. The number of surviving nematodes was counted daily until all nematodes were dead, determined by them no longer responding to the stimulation of the needle. This determination was performed in triplicate for each group.

### 2.9. Fertility Experiment

The grouping was the same as that described in Section 2.8. The groups were divided into XHT (L), XHT (H), YDBT (L), YDBT (H), XYMJ (L), XYMJ (H), and a control group, comprising a total of seven groups. One hundred microliters (100 μL) of the *E. coli* OP50 and sterile sample solution was distributed onto the 60 mm NGM plate sequentially (as described in Section 2.8). The synchronized nematodes at the L4 stage were transferred to the 60 mm NGM plate containing 150 μM 5-fluorouracil. For each group, one nematode was transferred. The day of the first transfer was recorded as day 0, and the nematodes were transferred to fresh plates every 24 h. The number of eggs laid by the nematodes was recorded daily from day 3 until the nematodes ceased laying eggs. This experiment was performed in triplicate for each group.

### 2.10. Measurement of ROS Levels

The grouping was the same as that mentioned in Section 2.8. The groups were divided into XHT (L), XHT (H), YDBT (L), YDBT (H), XYMJ (L), XYMJ (H), and a control group, making a total of seven groups. Five hundred microliters (500 μL) of the *E. coli* OP50 and equivalent volumes of sterile sample solution were distributed on the 90mm NGM plate sequentially. For each group, 1000 nematodes were prepared and incubated at a constant temperature and humidity for 3–5 days. After that, the nematodes in each group were separately collected in EP tubes and washed with 2 mL M9 buffer, followed by centrifugation at 1000 rpm for one minute. Subsequently, 500 microliters of nematode suspension was combined with 800 microliters of physiological saline and homogenized (60 Hz) for one minute in a tissue grinder. The ground nematode solution was subjected to centrifugation at 4000 rpm for two minutes. A total of 50 μL of supernatant in each group was added into the well of a black 96-well plate. DCFH-DA (100 μM) at a volume of 50 μL was then rapidly added. Fluorescence intensity was measured at 37 °C. The excitation and emission wavelengths were set at 485 nm and 538 nm. The measurement was performed every 5 min, and the experiment lasted for 2 h.

### 2.11. Measurement of Lipofuscin Levels

The grouping was the same as that described in Section 2.8. The groups were divided into XHT (L), XHT (H), YDBT (L), YDBT (H), XYMJ (L), XYMJ (H), and a control group, comprising a total of seven groups. Sample solutions at varying concentrations (100 μL) were plated onto 60 mm NGM agar plates (containing 5-fluorouracil) seeded with *E. coli* OP50, while sterile distilled water (100 μL) was used as the control. A total of 210 synchronized nematodes at the L4 stage were randomly divided into seven groups and transferred to the 60 mm NGM plate containing 150 μM 5-fluorouracil. Thirty nematodes were incubated for each group for 72 h and then collected in 2 mL EP tubes containing 2 mL of M9 buffer. The tubes were immersed in a water bath at 60 °C for 1 min and fixed on 2% agar slides. Fluorescence images were captured using an inverted fluorescence microscope. The fluorescence intensity of each image was quantified using ImageJ software. This measurement was performed in triplicate for each group.

### 2.12. Determination of Antioxidant Enzyme Activity and MDA Levels

Nematode supernatants were prepared as described in Section 2.10. The protein content of supernatants from each group was determined using a BCA kit, following the manufacturer’s instructions. The levels of relevant antioxidant enzymes, including SOD, CAT, and MDA, were determined using the SOD, CAT, and MDA detection kits, respectively. The final results were based on the total amount of protein measured in each group and expressed as U/mg prot, U/mg prot, and nmoL/mg prot.

### 2.13. Expression of Senescence Genes in Nematodes

The grouping was the same as that described in Section 2.8. Five hundred microliters (500 μL) of *E. coli* OP50 and equivalent volumes of sterile sample solution were sequentially distributed onto the 90 mm NGM plate containing 150 μM 5-fluorouracil. For each group, 1000 nematodes were prepared and incubated under constant temperature and humidity conditions for 5 days. After that, nematodes from each group were separately collected in EP tubes and washed with 2 mL of M9 buffer. After centrifugation at 1000 rpm for 1 min, the supernatant was discarded, and 1 mL of TRIzol solution was added and ground in a tissue grinder (60 Hz) for 1 min; this grinding step was repeated once. Then, the nematodes were collected, and the RNA was extracted by TRIzol. The extracted RNA was reverse transcribed to cDNA using the HiScript IV All-in-One Ultra RT SuperMix for qPCR (Vazyme, Nanjing, China) and primers designed by Sangong Biotech Co., Ltd, Shanghai, China (Table A1). The PCR reaction mixture (20 μL) contained 10 μL of 2*chamq UNIVERSAL SYBR QPCR MASTER MIX (Nanjing Vazyme Biotech Co., Ltd., Nanjing, China), 0.4 μL of forward primer (100 μM), 0.4 μL of reverse primer (100 μM), 2 μL CDNA, and 7.2 μL of ddH_2_O. Running in a fluorescence quantitative PCR instrument, the PCR System operated at 95 °C for 30 s, followed by 40 cycles of 95 °C for 10 s, 60 °C for 30 s, 95 °C for 15 s, 60 °C for 1 min, and 95 °C for 15 s. Genetic expression was analyzed using the comparative 2^−ΔΔCt^ method, with the actin-1 gene as the internal reference gene.

### 2.14. Statistics and Analysis

All data were presented as the mean ± standard deviation. Data and graphics were processed using GraphPad Prism 9.5. Values expressed as percentages (%) for the control group were normalized to 100%. Statistical analysis was performed by a one-way analysis of variance (ANOVA) or Student’s *t*-test. A significant difference was set at a *p*-value less than 0.5. The fertility, lipofuscin, and lifespan determination experiments used 90 nematodes per group with three parallels. The antioxidant enzyme determination and gene experiments used 1000–1200 nematodes per group. Each experiment was set with three repeated trials.

## 3. Results

### 3.1. Polysaccharide, Polyphenol, and Flavonoid Content of XHT Extracts

The polysaccharide, polyphenol, and flavonoid contents of the XHT and other tea samples (YDBT and XYMJ) were determined. The polysaccharide content of the XHT extracts was 23.0497 ± 0.3204 mg/g dry tea, which was similar to that of the XYMJ extracts (*p* > 0.05) but significantly higher than that of the YDBT extracts (*p* < 0.0001). Regarding the polyphenol and flavonoid contents, the XHT extracts contained 206.1709 ± 0.9456 mg/g dry tea and 5.1354 ± 0.2393 mg/g, respectively. Both contents were significantly higher than those of the XYMJ and YDBT extracts (*p* < 0.05) (Table 1).

### 3.2. Quantitative Analysis of Polyphenolic Component in XHT Extracts

Given that the XHT extracts possessed the highest polyphenol content among all tea extracts, we quantified the specific polyphenols in the XHT extracts using LC-MS/MS and identified the characteristic polyphenols by comparing them with the extracts from the other two kinds of tea.

**Figure 1:**
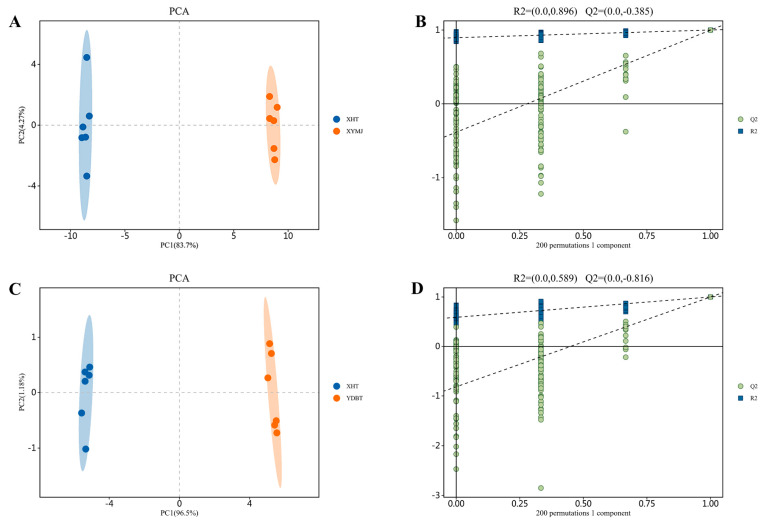
Multivariate statistical analyses of three different cultivar tea samples: (**A**,**C**) PCA score plot and (**B**,**D**) PLS-DA score plot.

The PCA plots (Figure 1A,C) show that there were discernible differences in the composition of the XHT, YDBT, and XYMJ extracts. The OPLS-DA model underwent 200 permutation tests (Figure 1B,D). The Q^2^ regression line intersected the vertical axis at a value less than 0, indicating that the model had not been overfitted. Thus, the model was validated, and the results were deemed useful for the identification and analysis of polyphenolic compounds in different teas.

**Figure 2:**
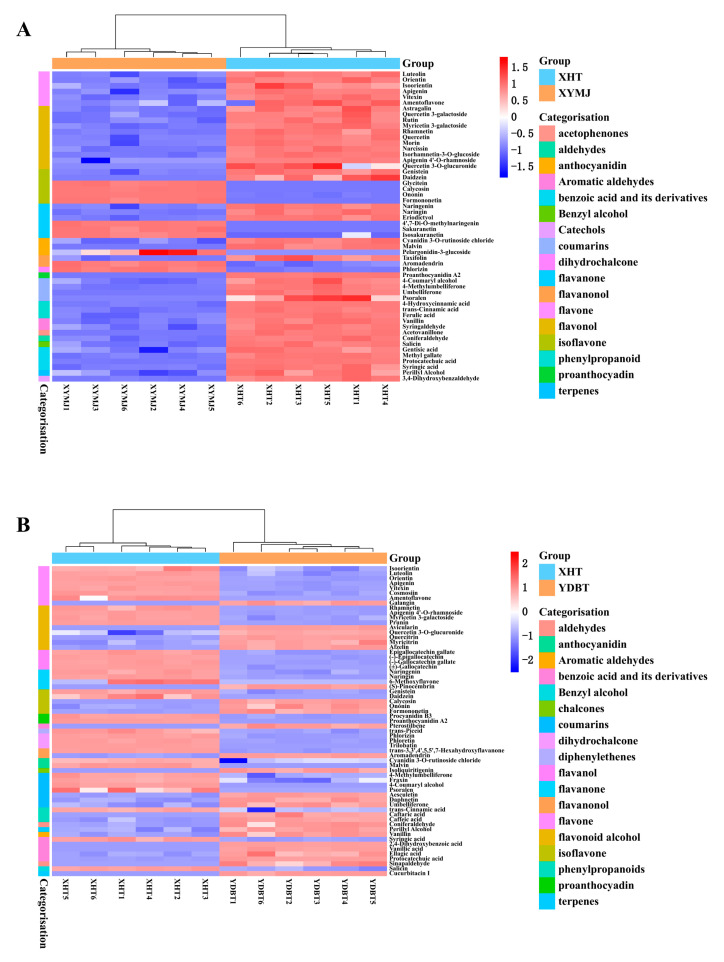
Heat-map showing the phenolic metabolite contents in water extracts of three types of tea. (**A**) Heatmap of differential metabolites between XHT and XYMJ. (**B**) Heatmap of differential metabolites between XHT and YDBT.

**Figure 3:**
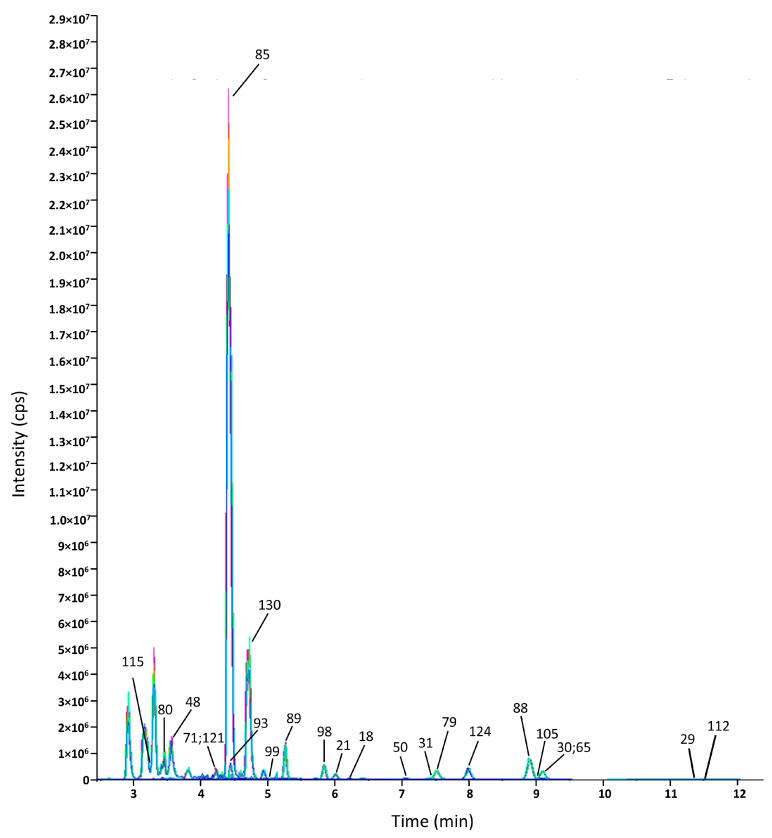
XIC (extracted ion chromatogram) diagrams of 23 characteristic phenolic compounds in XHT. The x-axis represents the retention time in minutes, while the y-axis shows the intensity in counts per second (cps).

Based on the multiplicity of differences FC ≥ 2, *p* < 0.05, and VIP > 1, 54 differential polyphenols of XHT and XYMJ (Figure 2A) and 63 differential polyphenols of XHT and YDBT (Figure 2B) were screened. The classification of differential substances was based on three principal categories: flavonoids, phenolic acids, and aroma substances. The flavonoid content of the XHT extracts was higher than that of the remaining two varieties, YDBT and XYMJ. A total of 90 differential metabolites were found from all three tea extracts—XHT, YDBT, and XYMJ—primarily including 57 flavonoids, 8 benzoic acids, and their derivatives, 5 phenylpropanoids, 2 aromatic aldehydes, and 9 coumarins. We further screened out 23 phenolic compounds whose content was significantly higher in the XHT extracts than in the XYMJ and YDBT extracts, which could have been the characteristic phenolics of the XHT extracts (Table 2). The numbers on the XIC diagrams (Figure 3) of the 23 characteristic phenolic compounds in XHT correspond to the substances listed in Table 2. Given the presence of compounds with relatively low concentrations among the 23 substances, individual XIC (Extracted Ion Chromatogram) plots for each compound are provided to facilitate comprehension (Figure S1–S4). Additionally, the peak areas and precursor-product ion pairs of the 23 characteristic phenolic compounds are presented in Table S1.

### 3.3. In Vitro Antioxidant Activity

**Figure 4:**
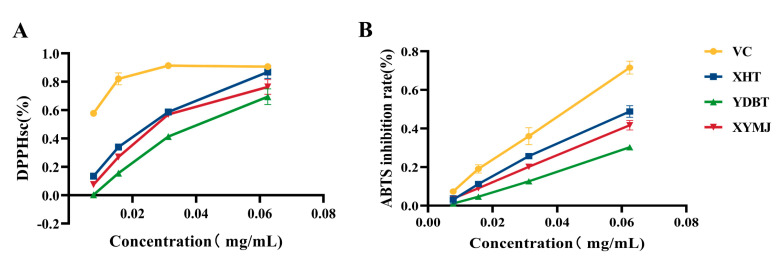
Determination of the ABTS-inhibitory capacity and DPPH-scavenging activity of XHT, TDBT, and XYMJ. (**A**) The DPPH-scavenging activity of XHT, TDBT, and XYMJ. (**B**) The ABTS-inhibitory capacity of XHT, TDBT, and XYMJ.

The DPPH clearance of the XHT extracts was 86.87 ± 0.05%, which was 10.5 ± 0.02% higher than that of the XYMJ extracts (*p* < 0.05) and 17.42 ± 0.07% higher than that of the YDBT extracts (*p* < 0.05) (Figure 3). Regarding the ABTS inhibition rate, the XHT extracts showed an inhibitory rate of 48.83 ± 0.03%, which was also 7.13 ± 0.03% higher than that of the XYMJ extracts (*p* < 0.05) and 18.55 ± 0.03% higher than that of the YDBT extracts (*p* < 0.05) (Figure 4). Taken together, both the DPPH-scavenging capacity and the ABTS-inhibitory rate of the XHT extracts were highest among all the tea samples, exhibiting the strongest antioxidant activity in vitro.

### 3.4. ROS and MDA Levels in Nematodes

**Figure 5:**
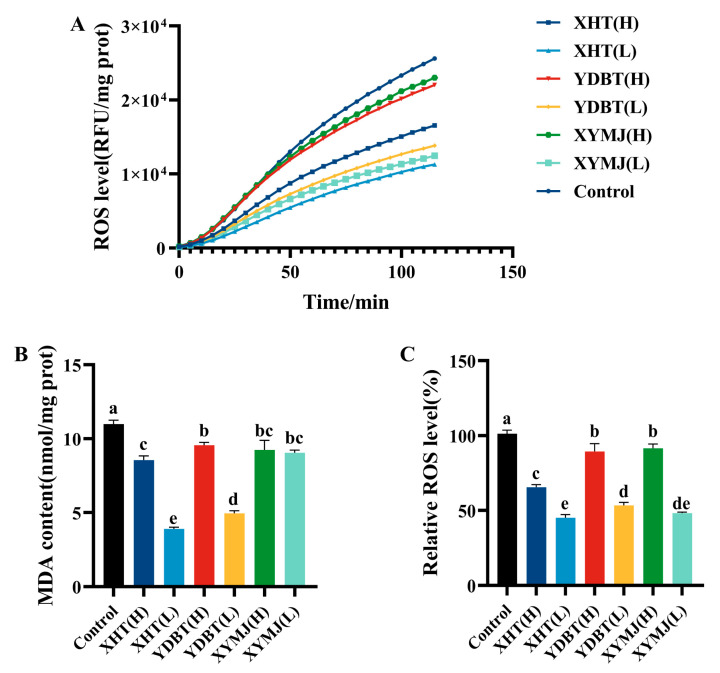
Effects of XHT on ROS and MDA content in *C. elegans*: (**A**) effect of XHT on ROS synthesis in *C. elegans*; (**B**) effect of XHT on the relative content of ROS in *C. elegans*; and (**C**) effect of XHT on MDA content in *C. elegans*. Different letters in the column are significantly different (*p* < 0.05).

The excessive production of reactive oxygen species (ROS) during the aging process may cause cellular damage and lead to the acceleration of aging [24,25]. In the nematode experiments, the intervention with the XHT extracts resulted in a significant reduction in the ROS levels at both low and high concentrations, with a mean decrease of 56.2 ± 3.99% and 35.76 ± 3.53%, respectively. The intervention using the other tea extracts was inferior. For example, the ROS content of the YDBT extracts at both low and high concentrations was 8.31 ± 1.43% and 44.26 ± 6.62% higher than that of XHT(L), while the ROS content of the XYMJ extracts at both low and high concentrations was 3.20 ± 0.25% and 46.36 ± 2.54% higher (Figure 5A,B). The XHT extracts exhibited the strongest ROS-reducing effect among all the extracts from tea samples. We further monitored the malondialdehyde (MDA) level in nematodes, as it was one of the markers reflecting oxidative damage caused by ROS [25,26]. The MDA content of the nematodes treated with the XHT extracts at both low and high concentrations was markedly diminished by 64.49 ± 0.04% (*p* < 0.05) and 22.12 ± 0.01% (*p* < 0.05) in comparison to the control (Figure 5C). Specifically, the MDA content in the nematodes treated with the lower concentration of the XHT extracts was 46.88 ± 0.03% (*p* < 0.05), 48.59 ± 0.07% (*p* < 0.05), 9.58 ± 0.02% (*p* < 0.05), and 51.54 ± 0.01% (*p* < 0.05) lower in groups XYMJ(L), XYMJ(H), YDBT(L), and YDBT(H), respectively. It can be concluded that the exposure of nematodes to the XHT extracts affected the prevention of ROS accumulation and the levels of MDA caused by oxidative stress.

### 3.5. Determination of the Content and Activity of Antioxidant Enzymes in Nematodes

A reduced ROS level likely accounts for the increased activity of antioxidant enzymes. SOD functions as a superoxide radical scavenger, while catalase (CAT), in the body, rapidly decomposes free radicals into water and oxygen. These enzymes collectively form a comprehensive antioxidant defense system [27]. Accordingly, the activities of two antioxidant enzymes, SOD and CAT, were determined in our study.

**Figure 6:**
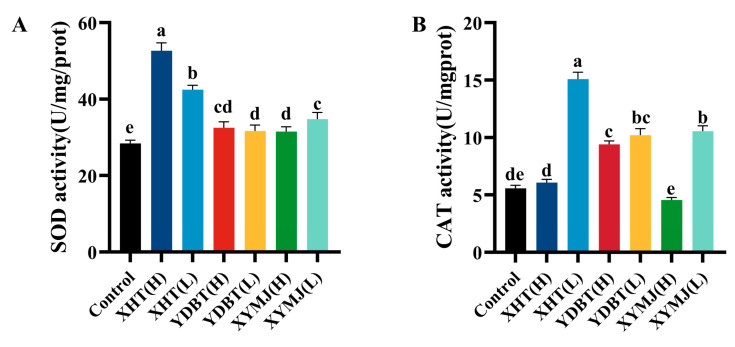
Effect of XHT, YDBT, and XYMJ on antioxidant enzymes in *C. elegans*: (**A**) effect of XHT, YDBT, and XYMJ on CAT antioxidant enzyme content in *C. elegans*; and (**B**) effect of XHT, YDBT, and XYMJ on SOD antioxidant enzyme content in *C. elegans*. Different letters in the column are significantly different (*p* < 0.05).

The CAT activity of the nematodes treated with the XHT extracts at a low concentration was markedly elevated by 170.95% (*p* < 0.05) in comparison to the control group, and this activity was 3.33, 1.43, 3.09, and 2.65 times higher than that of the nematodes treated with the XYMJ and YDBT extracts at both low and high concentrations (group XHT (L) versus groups XYMJ (L), XYMJ(H), YDBT (L), and YDBT (H), respectively) (*p* < 0.05) (Figure 6B).

Additionally, the SOD activity of the nematodes treated with the XHT extracts at high and low concentrations was markedly elevated by 85.53% and 49.50%, respectively, in comparison to the control (*p* < 0.05) (Figure 6A). It was significantly higher than that of the nematodes treated with the other tea sample extracts. For example, when compared with the SOD activity of the nematodes treated with the XHT extracts at the high concentration, the SOD activity of the nematodes treated with the XYMJ and YDBT extracts at both low and high concentrations was 63.09%, 74.53%, 74.00%, and 71.08% higher, respectively (*p* < 0.05). The treatment employing the XHT extracts resulted in the increase in both the amount and activity of antioxidant enzymes, therefore reducing the adverse effect of ROS.

### 3.6. Effects on Lipofuscin Levels in Nematodes

**Figure 7:**
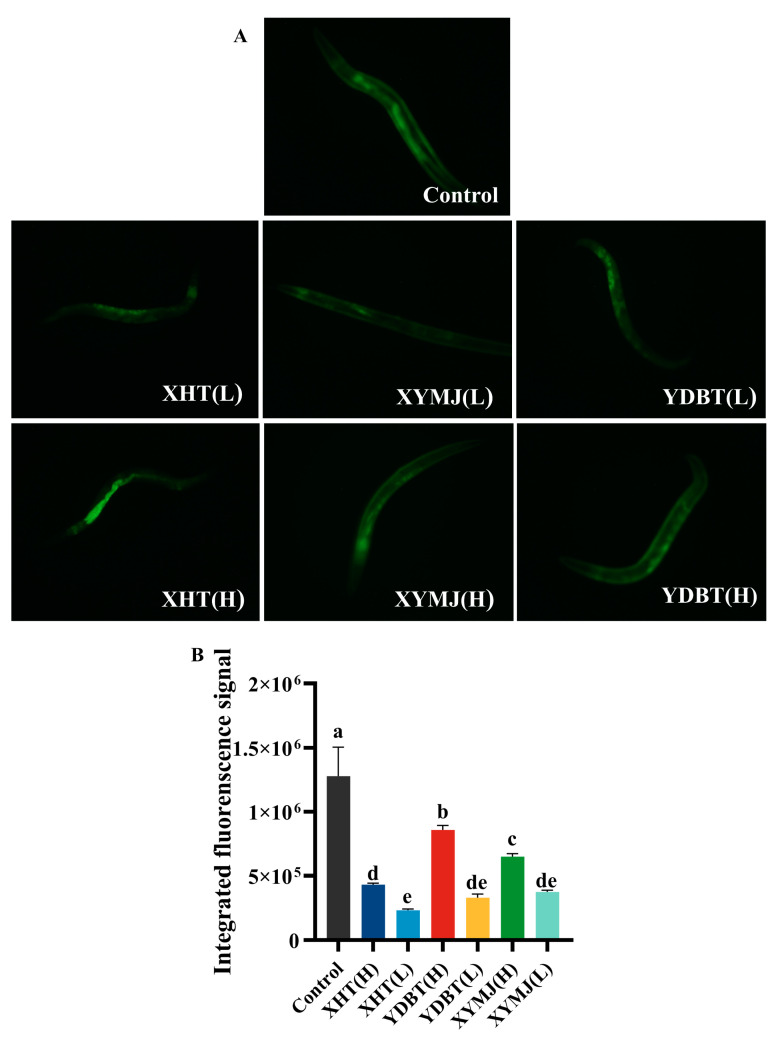
Effect of XHT on lipofuscin accumulation in *C. elegans*: (**A**) fluorescence of lipofuscin in *C. elegans* and (**B**) quantitative determination of lipofuscin by Image J 1.45. Different letters in the column are significantly different (*p* < 0.05).

Lipofuscin is a biomarker of the aging process [28]. Prior research has demonstrated that lipofuscin accumulates in nematodes with aging, causing fluorescence to increase gradually over time [29]. The fluorescence intensity of various treatment groups was markedly diminished in comparison to that of the control group (Figure 7). All treatments resulted in a concentration-dependent reduction in lipofuscin accumulation in the nematodes, with the XHT extracts having the best performance. Following the XHT treatment at both low and high concentrations, the lipofuscin content in the nematodes was found to decrease by 81.85% and 66.14% compared to the control group, respectively. Moreover, the lipofuscin levels of the nematodes treated with the XHT extract at a low concentration were 11.24% and 32.63% lower than those treated with the XYMJ extracts at the low and high concentrations and 7.77% and 48.98% lower than those treated with the YDBT extracts at the low and high concentration, respectively. It can be inferred that the anti-aging effect of the XHT extract in nematodes can be attributed to its ability to reduce the formation of lipofuscin.

### 3.7. Effects on Lifespan Extension and Reproductive Toxicity

**Figure 8:**
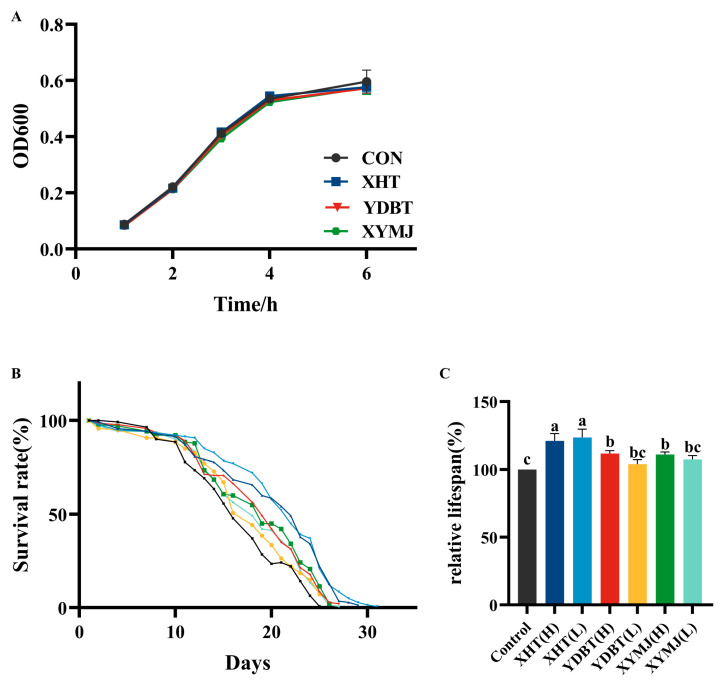
(**A**) Effects of different samples on the growth of *E. coli* OP50. (**B**) Effect of XHT on the lifespan of *C. elegans*. (**C**) Impact of XHT on the relative lifespan of *C. elegans*. Values with different letters in the column are significantly different (*p* < 0.05).

As one of the quantitative indications of aging, the effect of the tea samples, especially the XHT extracts, on lifespan was assessed [30]. Under experimental conditions, we found that the lifespan of the nematodes in the control group was 15.66 ± 0.17 days. The lifespan of the nematodes treated with the XHT extract at the low and high concentrations was 19.34 ± 0.83 and 18.96 ± 0.72 days, respectively. This lifespan was significantly longer than that of the nematodes in the control group, by 23.50% and 21.07%. By comparison, the nematodes treated with the XYMJ extract at the low and high concentrations exhibited a lifespan of 16.80 ± 0.40 and 17.37 ± 0.26 days, respectively, and those treated with the YDBT extracts at the low and high concentrations showed a lifespan of 16.29 ± 0.43 and 17.49 ± 0.28 days, respectively. Statistical analysis revealed that the lifespan of the nematodes treated with the XHT extracts at the low concentration was similar to that measured at the high concentration (*p* > 0.05). Still, both of them were significantly longer than those of the nematodes treated with the other tea extracts (*p* < 0.05). Hence, treatment with the XHT extracts significantly prolonged the lifespan of the nematodes (Figure 8B,C).

**Figure 9:**
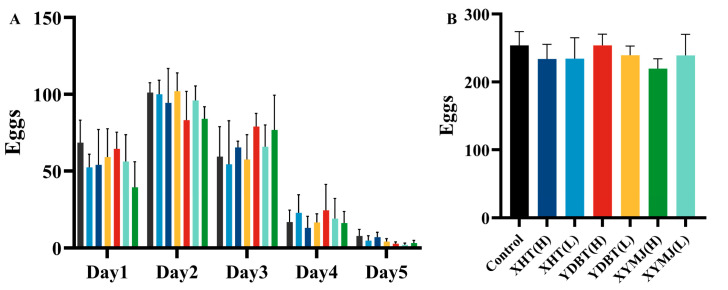
Effect of XHT on oviposition ability of *C. elegans*: (**A**) daily egg production of *C. elegans* and (**B**) effect of XHT on total oviposition of *C. elegans*.

Previous studies have shown that reduced reproduction is associated with a longer lifespan [27]. We therefore examined whether the tea samples, especially the XHT extracts, had a reproductive toxicity. The average number of eggs produced in the control group (without any tea extract treatment) was 253.8 ± 18.07. The nematodes laid 234.4 ± 27.59 and 233.8 ± 19.37 eggs after treatment with the XHT extracts at low and high concentrations, respectively. There was no significant difference in egg production between the nematodes treated with the XHT, XYMJ, and YDBT extracts (*p* > 0.05) (Figure 9). Other studies also documented how dietary restriction increased the lifespan and healthspan across *C. elegans* [31]. To determine whether the XHT extracts could influence the lifespan and other physiological processes of the nematodes by limiting their dietary intake, we assessed the growth of *E. coli* OP50. As shown in Figure 8A, the growth curves of *E. coli* OP50 treated with the extracts of XHT, YDBT, and XYMJ extracts exhibited a high degree of overlap with the control group. This result suggested that the three aqueous extracts of tea did not exert any inhibitory effects on the growth of *E. coli* OP50. Therefore, the XHT extract treatment prolonged the lifespan of the nematodes without reproductive toxicity and dietary restriction.

### 3.8. Effect of Phenolic Compounds in XHT Extracts on Anti-Aging Gene Expression

**Figure 10:**
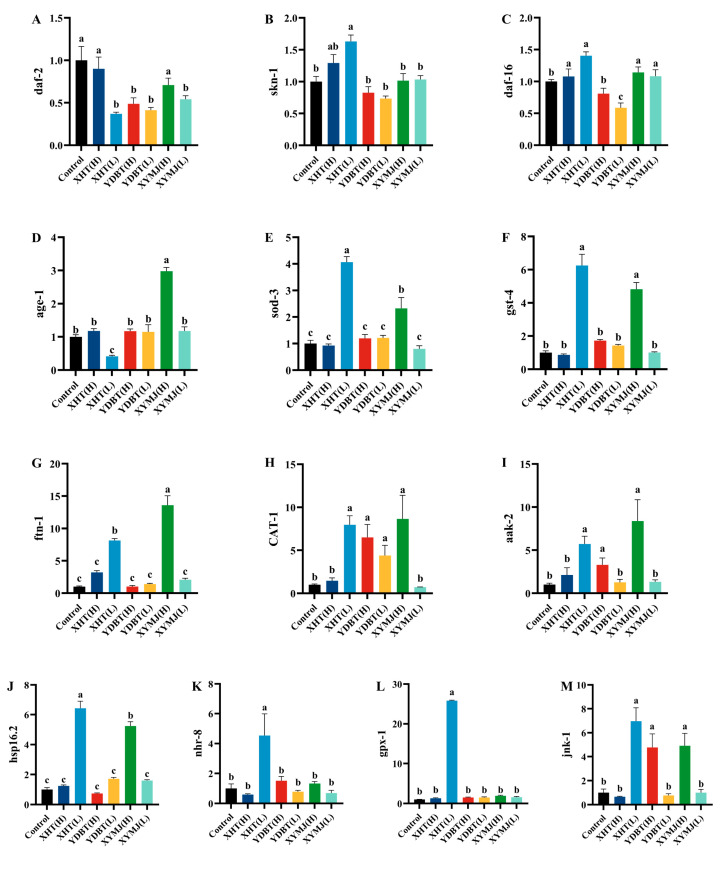
Relative expression levels of anti-aging-related genes in *C. elegans* after treatment with XHT. Different letters in the column are significantly different (*p* < 0.05). (**A**–**M**) The expression levels of genes *daf-2*, *skn-1*, *daf-16*, *age-1*, *sod-3*, *gst-4*, *ftn-1*, *cat-1*, *aak-2*, *hsp16.2*, *nhr-8*, *gpx-1* and *jnk-1*, respectively.

**Figure 11:**
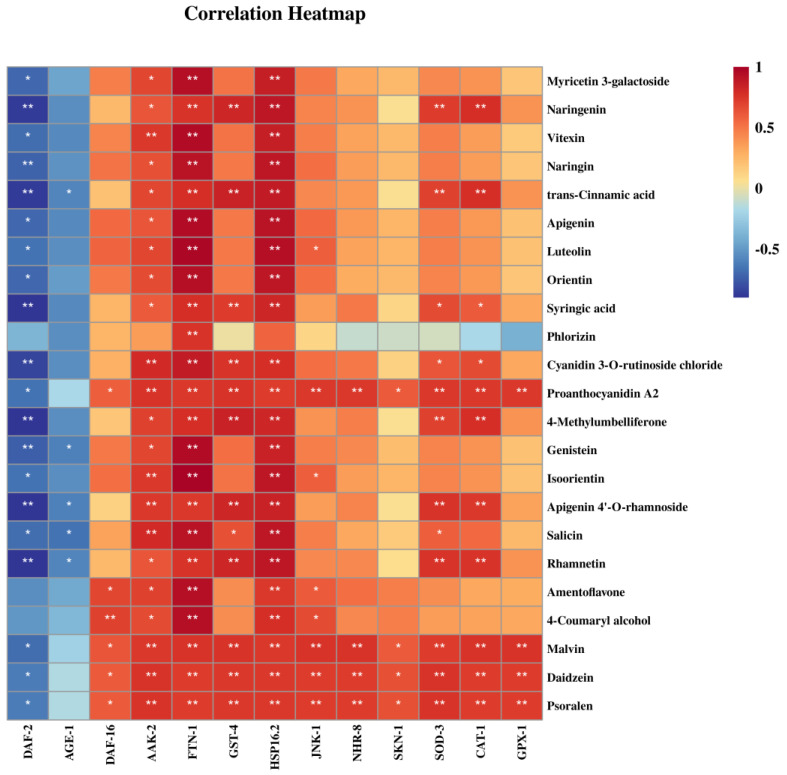
Correlation analysis between characteristic phenolic compounds and aging-related genes in XHT. Asterisks (*) in the correlation analysis are considered to be significantly different (* *p* < 0.05 and ** *p* < 0.01).

Previous reports have indicated that aging is linked to multiple signaling pathways, including the target of the autophagy-related (TOR) signaling pathway of rapamycin, the insulin/insulin-like growth factor 1 (IGF-1) signaling (IIS) pathway, the mitochondria-associated functional signaling pathway, and the adenosine monophosphate (AMP)-activated protein kinase (AMPK) signaling pathway [10]. Therefore, we determined the mRNA levels of the genes associated with these pathways. Gene expression in the control group was defined as 1. We found that the mRNA levels of genes *daf-2* and *age-1* in the nematodes treated with the low-concentration XHT extracts were significantly lower than those treated with the XYMJ extracts, YDBT extracts, and ddH_2_O (the control group) (*p* < 0.05), while the mRNA contents of genes *sod-3*, *skn-1*, *nhr-8*, and *hsp16.2* were significantly higher (*p* < 0.05). In addition, the mRNA contents of genes *daf-16*, *gst-4*, *aak-2*, and *jnk-1* in the nematodes treated with the XHT extracts at the low concentration were similar to those treated with the XYMJ extracts at the high concentration (Figure 10), but their contents were higher than those of the nematodes in the control group. GPX-1 is a member of the GPX family, one of the most important components of the antioxidant system, involved in the major antioxidant molecule glutathione [32]. After treatment with the XHT extracts at a low concentration, the mRNA content of the *gpx-1* gene was approximately 16.7 times higher than that in the other treatment groups as well as the control group (*p* < 0.05) (Figure 10M). Correlation analysis further illustrated the relationship between the modulation of anti-aging-related genes by the XHT extracts and the potential characteristic phenolic compounds in the XHT extracts. We found that all 23 compounds were negatively correlated with the expression of the *daf-2* gene and positively correlated with the expression of most genes (Figure 11).

## 4. Discussion

The chemical components of tea, particularly its phenolic compounds, vary depending on factors such as cultivation methods, processing techniques, tea varieties, and the growing environment [7,8]. This study represents the first comprehensive analysis of the phenolic compounds in the aqueous extracts of Xianhu tea (XHT), a renowned tea from Guangdong, China. The principal phenolic compounds identified in the XHT extracts were predominantly catechins, flavonoids, and phenolic acids. The concentration of certain flavonoids, including rutin, epicatechin, gallic acid, nicotiflorin, astragalin, (-)-epicatechin 3-O-gallate, and (±) -catechin, was notably elevated. The predominant class among these substances was that of catechins, which are ubiquitous in tea and found in particularly high concentrations in tea seed extracts and green tea [33,34]. Additionally, the analysis revealed that 23 phenolic compounds, including myricetin 3-galactoside, vitexin, naringenin, naringin, trans-cinnamic acid, luteolin, and orientin, were present in significantly higher amounts in XHT compared to two other teas (XYMJ and YDBT). These compounds were thus proposed to be characteristic phenolics of XHT. Relevant studies have demonstrated that the polyphenol content in white tea is 22.90%, which is almost identical to that in XHT. This level of polyphenol content has been proven to possess excellent antioxidant and anti-aging properties, significantly influencing the lifespan of fruit flies and modulating the expression of related genes [35]. Polyphenols from Blumea laciniata extend the lifespan of nematodes to 19.17 ± 1.61 days by modulating the IIS pathway [36], while XHT increases the lifespan to 19.34 ± 0.83 days, showing a slightly superior effect compared to polyphenols from Blumea laciniata. Genistein, 4-coumaryl alcohol, amentoflavone, and psoralen are not commonly found in green tea, and research on them is relatively limited. Malvin, an anthocyanin present in green tea, has also not been extensively studied. As characteristic phenolic compounds in XHT, they may be among the reasons for its superior anti-aging effects.

Tea is recognized as a rich source of a variety of polyphenols, including flavanols, flavonoids, and phenolic acids [37], making it an ideal source for the development of tea polyphenol functional foods [38]. Tea polyphenols have been demonstrated to inhibit oxidative enzymes by quenching free radicals, thereby reducing the production of free radicals and enhancing antioxidant enzyme activities [39]. The XHT extracts in our study showed strong in vitro antioxidant effects, significantly reduced the ROS and MDA levels in *C. elegans*, and increased the activity of antioxidant enzymes, such as catalase (CAT) and superoxide dismutase (SOD). These effects were accompanied by a reduction in lipofuscin accumulation and an extension of lifespan in the nematodes, achieved without reproductive toxicity or dietary restriction. It was demonstrated that myricetin and its derivatives enhanced antioxidant enzyme activity and conferred anti-aging properties [40,41,42,43]. In other studies, apigenin has been demonstrated to inhibit the process of cellular senescence, thereby reducing the risk of age-related cardiovascular disease [44,45]. Additionally, it has been shown to significantly extend the lifespan of nematodes [46]. Furthermore, research has demonstrated the antioxidant and anti-aging properties of vitexin, naringenin, naringin, luteolin, and orientin [47,48,49,50]. These findings suggest that the characteristic phenolics present in XHT are responsible for the tea’s beneficial effects in terms of antioxidant activity and mitigating aging.

Thirteen aging-associated genes, such as *daf-2*, *age-1*, *daf-16*, *sod-3*, *hsp16.2*, *skn-1,* and *akk-2* etc., were further detected. The expression of these genes was significantly altered following treatment with the XHT extracts. Correlation analysis revealed that the expression levels of these 13 aging-related genes were significantly correlated with the 23 potential characteristic phenolic compounds. Previous reports indicated that astragalin synergistically extended the lifespan of *C. elegans* by upregulating the expression of *daf-16* through inhibiting the expression of daf-2/IGFR and also activating the AMPK and MAPK pathways to upregulate the expression of *sir-2.1, sir-2.4*, and *skn-1* [51]. Myricetin and its derivatives were observed to enhance the thermal stress and oxidative stress characteristics of nematodes and extend their lifespan. This longevity effect was dependent on *daf-16* and not attributable to the antioxidant effect of flavonoids [43,52]. Vitexin has been demonstrated to prolong the lifespan of nematodes by enhancing the expression of the *sod-3* and *hsp-16.2* genes [53]. Naringenin upregulated the expression levels of *daf-16*, *sek-1,* and *skn-1*, downregulated the expression levels of *daf-2* and *age-1*, and further activated *sod-3* and *gst-4* [50]. The administration of lignans and naringin was found to extend the lifespan of normal nematodes but not the *aak-2*-knockout nematodes, indicating that the lifespan extension was attributed to the regulation of the *aak-2* gene [54].

In summary, the effects of the XHT extracts were superior to those of other teas in reducing oxidative stress, ROS levels, lipofuscin accumulation, and MDA levels, significantly enhancing SOD and CAT activities, modulating aging-related gene expression, and extending lifespan in nematodes. These effects are likely due to the 23 phenolic compounds present in significantly higher concentrations in XHT compared to XYMJ and YDBT. These phenolics are likely the primary contributors to XHT’s anti-aging effects and may represent its characteristic bioactive components.

This study demonstrates the superior antioxidant and anti-aging effects of XHT compared to XYMJ and YDBT. These 23 phenolic compounds, including myricetin, vitexin, naringenin, and apigenin, likely contribute to these benefits. The XHT extracts not only reduced the ROS and MDA levels but also enhanced SOD and CAT activity, reduced lipofuscin accumulation, and extended the lifespan of the nematodes. The modulation of aging-related genes further supports the mechanistic role of these phenolics in XHT’s bioactivity.

## 5. Conclusions

Our findings indicated that the Xianhu tea (XHT) extracts had significantly higher concentrations of polyphenols than the other two tea extracts, Xinyang Mao Jian tea (XYMJ) and Yingde black tea (YDBT), both which are commonly consumed in China. Specifically, the 23 types of phenolic compounds that were found in the highest quantities in the XHT extracts could be considered as the potential characteristic phenolics of the XHT extracts. Further comparative analysis revealed that the XHT extracts exhibited the best performance in DPPH clearance and ABTS inhibition, the strongest capacity to reduce ROS, MDA, and lipofuscin levels, and the most significant enhancement in the expression of SOD and CAT. All these effects resulted in the longest extension of lifespan in *C. elegans*. Genes associated with the aging process, including *daf-2*, *age-1*, *daf-16*, *gst-4*, *sod-3*, *hsp16.2*, *cat-1*, *gpx-1*, *skn-1*, *jnk-1*, *aak-2*, *ftn-1*, and *nhr-8*, were also modulated by the XHT extracts, and the 23 potential characteristic phenolics were likely the key substances responsible for the anti-aging effect of the XHT extracts.

The aqueous extract of Xianhu tea (XHT) and the phenolic compounds within it may hold potential for the development of anti-aging and antioxidant dietary supplements, such as tea beverages and health products. The discovery of the anti-aging effects of the XHT extract has paved a promising way for its application in the healthcare, pharmaceutical, and food industries. Among the 23 characteristic phenols identified in this study, there are components that are currently under-researched and warrant further investigation. Future research should further investigate how the characteristic phenolic compounds in XHT water extracts regulate aging-related pathways and whether synergistic effects exist among multiple phenolic compounds. These efforts aim to deepen our understanding of the underlying mechanisms and further optimize the application of the XHT water extract and its specific components.

There might have been certain biases in the sample selection for this study. For example, the small sample size or limited sample sources may have restricted the generalizability of the research findings. The experimental results may have also been influenced by external variables, such as the experimental instruments used, the experimental methods adopted, and the duration of the experiment. Moreover, human operational errors may have also taken place.

## Figures and Tables

**Table 1:** Polysaccharide, polyphenol, and flavonoid content of tea aqueous extracts (mean ± SD).

Group	Polysaccharide Content (mg/g Dry Tea ± SD)	Polyphenol Content (mg/g Dry Tea ± SD)	Flavonoid Content (mg/g Dry Tea ± SD)
XHT	23.0497 ± 0.3204 ^a^	206.1709 ± 0.9456 ^a^	5.1354 ± 0.2393 ^a^
YDBT	19.6990 ± 0.5782 ^b^	133.3794 ± 2.0122 ^c^	4.0187 ± 0.1303 ^c^
XYMJ	22.4382 ± 0.1394 ^a^	166.9787 ± 1.1612 ^b^	4.6300 ± 0.7343 ^b^

Noted: Different letters in lowercase in the same column indicate significant differences between different groups (*p* < 0.05).

**Table 2:** Characteristic phenols of XHT, YDBT, and XYMJ (mean ± SD).

Metabolites (Number)	XHT (ng/mL)	XYMJ (ng/mL)	YDBT (ng/mL)
Luteolin (79)	3343.57 ± 130.62 ^a^	1469.33 ± 100.78 ^b^	592.86 ± 117.75 ^c^
Orientin (93)	2911.33 ± 135.48 ^a^	1261.73 ± 85.38 ^b^	376.61 ± 24.80 ^c^
Isoorientin (71)	329.39 ± 56.91 ^a^	106.47 ± 15.37 ^b^	86.04 ± 19.08 ^b^
Apigenin (30)	5248.16 ± 186.58 ^a^	1675.53 ± 243.92 ^b^	573.83 ± 40.22 ^c^
Vitexin (130)	14,242.06 ± 1027.54 ^a^	3039.50 ± 312.81 ^b^	1518.14 ± 101.19 ^c^
Amentoflavone (29)	26.01 ± 11.26 ^a^	5.16 ± 1.53 ^b^	0 ^b^
Myricetin 3-galactoside (85)	66,786.83 ± 3798.76 ^a^	22,854.64 ± 1430.50 ^b^	8251.01 ± 1317.31 ^c^
Rhamnetin (112)	175.72 ± 13.23 ^a^	54.61 ± 1.64 ^b^	60.16 ± 1.29 ^b^
Apigenin 4′-O-rhamnoside (31)	2020.02 ± 5.76 ^a^	22.12 ± 8.02 ^b^	28.98 ± 1.11 ^b^
Genistein (65)	449.71 ± 48.76 ^a^	68.13 ± 9.11 ^b^	41.45 ± 7.47 ^b^
Daidzein (50)	5.36 ± 2.12 ^a^	0 ^b^	0 ^b^
Naringenin (88)	18,631.95 ± 672.69 ^a^	7762.99 ± 618.52 ^b^	8990.59 ± 565.74 ^b^
Naringin (89)	13,505.05 ± 608.34 ^a^	5510.38 ± 151.54 ^b^	1731.10 ± 83.88 ^c^
Cyanidin 3-O-rutinoside chloride (48)	1393.81 ± 67.91 ^a^	450.03 ± 65.09 ^b^	598.77 ± 200.29 ^b^
Malvin (80)	12.76 ± 1.69 ^a^	0 ^b^	0 ^b^
Phlorizin (98)	1676.33 ± 28.19 ^a^	4369.75 ± 103.26 ^b^	704.02 ± 21.95 ^c^
Proanthocyanidin A2 (99)	1271.09 ± 134.51 ^a^	0 ^b^	0 ^b^
4-Coumaryl alcohol (18)	20.40 ± 1.46 ^a^	9.82 ± 0.78 ^b^	0 ^b^
4-Methylumbelliferone (21)	573.53 ± 21.92 ^a^	213.59 ± 7.19 ^b^	262.79 ± 34.21 ^b^
Psoralen (105)	1.25 ± 0.55 ^a^	0 ^b^	0 ^b^
Trans-cinnamic acid (124)	8406.17 ± 295.10 ^a^	2376.58 ± 117.05 ^b^	3927.76 ± 881.13 ^c^
Salicin (115)	167.18 ± 7.46 ^a^	45.26 ± 4.99 ^b^	44.07 ± 5.60 ^b^
Syringic acid (121)	1440.59 ± 209.19 ^a^	115.81 ± 9.91 ^b^	110.58 ± 10.92 ^b^

Noted: Different letters in lowercase in the same line indicate significant differences between different groups (*p* < 0.05). The larger the value of R^2^, the better the model fit.

## Data Availability

The original contributions presented in the study are included in the article, further inquiries can be directed to the corresponding author.

## Supplementary Materials

The following supporting information can be downloaded at: https://www.mdpi.com/article/10.3390/foods14050737/s1, Due to the presence of low-abundance compounds among the 23 phenolic compounds in XHT, which were not clearly displayed in Figure 3, additional individual XIC plots and information on peak areas and precursor-product ion pairs are provided for clarity. The x-axis represents the retention time in minutes, while the y-axis shows the intensity in counts per second (cps). Figure S1: XIC (Extracted Ion Chromatogram) plots of six phenolic compounds in XHT, namely 4-Coumaryl alcohol, Luteolin, Vitexin, Apigenin, Naringenin, and Rhamnetin. Figure S2: XIC (Extracted Ion Chromatogram) plots of six compounds in XHT, namely Phlorizin, Salicin, Apigenin 4′-O-rhamnoside, Orientin, Amentoflavone, and Isoorientin. Figure S3: XIC (Extracted Ion Chromatogram) plots of six compounds in XHT, namely Proanthocyanidin A2, trans-Cinnamic acid, Naringin, 4-Methylumbelliferone, Syringic acid, and Psoralen. Figure S4: XIC (Extracted Ion Chromatogram) plots of five compounds in XHT, namely Malvin, Cyanidin 3-O-rutinoside chloride, Myricetin 3-galactoside, Genistein, and Daidzein. Table S1: Peak areas and precursor-product ion pairs of the 23 characteristic phenolic compounds in XHT.

## Abbreviations

XHT	Xianhu tea water extract
XYMJ	Xinyang Maojian tea water extract
YDBT	Yingde black tea water extract
DPPH	2,2-diphenyl-1-picrylhydrazyl
ABTS	2,2′-azinobis-(3-ethylbenzthiazoline-6-sulfonic acid)
*C. elegans*	*Caenorhabditis elegans*
5-FUDR	5-fluorouracil

## Appendix A

**Table A1:** Genetic sequence.

Gene	Direction	Primer Sequences (5′-3′)
*actin-1*	Forward	TCGGTATGGGACAGAAGGAC
	Reverse	CATCCCAGTTGGTGACGATA
*ftn-1*	Forward	CGAGTGGGGAACTGTCCTTG
	Reverse	TCATTGATCGAATGTACCTGCTCT
*daf-16*	Forward	TCCGTCACAATCTGTCTCTTCATTC
	Reverse	GTGTACGCCGTGGATTCCTTC
*age-1*	Forward	CCTGAACCGACTGCCAATC
	Reverse	GTGCTTGACGAGATATGTGTATTG
*sod-3*	Forward	CAACTTGGCTAAGGATGGTGGAG
	Reverse	AGCCTTGAACCGCAATAGTGATG
*skn-1*	Forward	GGTCTCCGTTGGCGTGATGATC
	Reverse	CTGGTGGATGCTCGGTGAGTATTG
*aak-2*	Forward	ACCTCTCGCTCTTCCGCCATC
	Reverse	TCAGCCGTCCGTGCTTAACAATG
*gst-4*	Forward	AGTTGTTGAACCAGCCCGTGATG
	Reverse	GCCCAAGTCAATGAGTCTCCAACG
*nhr-8*	Forward	GTCTCGAAGGTCTGCTGTTCCAC
	Reverse	CGCCTTACAAGATTCGCAAGTGAG
*hap16.2*	Forward	GTAGATGTTGGTGCAGTTGCTTCG
	Reverse	CCTTGAACCGCTTCTTTCTTTGGC
*jnk-1*	Forward	ACACTCTGCTCGCATCCTCCTC
	Reverse	CAGCCAATTCCCAACGGACTCG
*gpx-1*	Forward	GTCACTTTCGGATTACAAAGGAAA
	Reverse	GGGAAGGCAAGAACTTCGAGA
*cat-1*	Forward	CCAGTTGGACGAAGTGAGAGGAG
	Reverse	AGGTTTCTTGGCAGCAGGAGAC

## References

[1] Tang G.-Y., Meng X., Gan R.-Y., Zhao C.-N., Liu Q., Feng Y.-B., Li S., Wei X.-L., Atanasov A.G., Corke H. (2019). Health Functions and Related Molecular Mechanisms of Tea Components: An Update Review. Int. J. Mol. Sci..

[2] Khan N., Mukhtar H. (2018). Tea Polyphenols in Promotion of Human Health. Nutrients.

[3] Xing L., Zhang H., Qi R., Tsao R., Mine Y. (2019). Recent Advances in the Understanding of the Health Benefits and Molecular Mechanisms Associated with Green Tea Polyphenols. J. Agric. Food Chem..

[4] Chowaniak M., Niemiec M., Zhu Z., Rashidov N., Gródek-Szostak Z., Szeląg-Sikora A., Sikora J., Kuboń M., Fayzullo S.A., Mahmadyorzoda U.M. (2021). Quality Assessment of Wild and Cultivated Green Tea from Different Regions of China. Molecules.

[5] Zheng X.-Q., Li Q.-S., Xiang L.-P., Liang Y.-R. (2016). Recent Advances in Volatiles of Teas. Molecules.

[6] Zhang Q., Li T., Wang Q., LeCompte J., Harkess R.L., Bi G. (2020). Screening Tea Cultivars for Novel Climates: Plant Growth and Leaf Quality of *Camellia sinensis* Cultivars Grown in Mississippi, United States. Front. Plant Sci..

[7] Ye J., Wang Y., Kang J., Chen Y., Hong L., Li M., Jia Y., Wang Y., Jia X., Wu Z. (2022). Effects of Long-Term Use of Organic Fertilizer with Different Dosages on Soil Improvement, Nitrogen Transformation, Tea Yield and Quality in Acidified Tea Plantations. Plants.

[8] Ge S., Wang Y., Shen K., Wang Q., Ahammed G.J., Han W., Jin Z., Li X., Shi Y. (2024). Effects of Differential Shading on Summer Tea Quality and Tea Garden Microenvironment. Plants.

[9] Luo J., Mills K., le Cessie S., Noordam R., van Heemst D. (2020). Ageing, Age-Related Diseases and Oxidative Stress: What to Do Next?. Ageing Res. Rev..

[10] Liu L., Guo P., Wang P., Zheng S., Qu Z., Liu N. (2021). The Review of Anti-Aging Mechanism of Polyphenols on *Caenorhabditis elegans*. Front. Bioeng. Biotechnol..

[11] Duan H., Pan J., Guo M., Li J., Yu L., Fan L. (2022). Dietary Strategies with Anti-Aging Potential: Dietary Patterns and Supplements. Food Res. Int..

[12] Wu M., Cai J., Fang Z., Li S., Huang Z., Tang Z., Luo Q., Chen H. (2022). The Composition and Anti-Aging Activities of Polyphenol Extract from *Phyllanthus emblica* L. Fruit. Nutrients.

[13] Luo J., Si H., Jia Z., Liu D. (2021). Dietary Anti-Aging Polyphenols and Potential Mechanisms. Antioxidants.

[14] Shen P., Yue Y., Zheng J., Park Y. (2018). *Caenorhabditis elegans*: A Convenient In Vivo Model for Assessing the Impact of Food Bioactive Compounds on Obesity, Aging, and Alzheimer’s Disease. Annu. Rev. Food Sci. Technol..

[15] Shen P., Yue Y., Park Y. (2018). A Living Model for Obesity and Aging Research: *Caenorhabditis elegans*. Crit. Rev. Food Sci. Nutr..

[16] Lin Y., Lin C., Cao Y., Chen Y. (2023). *Caenorhabditis elegans* as an in Vivo Model for the Identification of Natural Antioxidants with Anti-Aging Actions. Biomed. Pharmacother..

[17] Ghasemzadeh A., Jaafar H.Z.E., Rahmat A. (2010). Antioxidant Activities, Total Phenolics and Flavonoids Content in Two Varieties of Malaysia Young Ginger (*Zingiber officinale* Roscoe). Molecules.

[18] He M., Zeng J., Zhai L., Liu Y., Wu H., Zhang R., Li Z., Xia E. (2017). Effect of in Vitro Simulated Gastrointestinal Digestion on Polyphenol and Polysaccharide Content and Their Biological Activities among 22 Fruit Juices. Food Res. Int..

[19] Tian Z.-X., Li Y.-F., Long M.-X., Liang Q., Chen X., Huang D.-M., Ran Y.-Q. (2023). Effects of Six Different Microbial Strains on Polyphenol Profiles, Antioxidant Activity, and Bioaccessibility of Blueberry Pomace with Solid-State Fermentation. Front. Nutr..

[20] Zhong B., Robinson N.A., Warner R.D., Barrow C.J., Dunshea F.R., Suleria H.A.R. (2020). LC-ESI-QTOF-MS/MS Characterization of Seaweed Phenolics and Their Antioxidant Potential. Mar. Drugs.

[21] Berger S., Oesterle I., Ayeni K.I., Ezekiel C.N., Rompel A., Warth B. (2024). Polyphenol Exposure of Mothers and Infants Assessed by LC-MS/MS Based Biomonitoring in Breast Milk. Anal. Bioanal. Chem..

[22] García A., González Alriols M., Spigno G., Labidi J. (2012). Lignin as Natural Radical Scavenger. Effect of the Obtaining and Purification Processes on the Antioxidant Behaviour of Lignin. Biochem. Eng. J..

[23] Wei Q., Zhang Y.-H. (2023). Ultrasound-Assisted Polysaccharide Extraction from *Cercis chinensis* and Properites, Antioxidant Activity of Polysaccharide. Ultrason. Sonochem.

[24] Xiao H., Jedrychowski M.P., Schweppe D.K., Huttlin E.L., Yu Q., Heppner D.E., Li J., Long J., Mills E.L., Szpyt J. (2020). A Quantitative Tissue-Specific Landscape of Protein Redox Regulation during Aging. Cell.

[25] Zhang C., Song X., Cui W., Yang Q. (2021). Antioxidant and Anti-Ageing Effects of Enzymatic Polysaccharide from *Pleurotus eryngii* Residue. Int. J. Biol. Macromol..

[26] Wu H., Kong Y., Zhao W., Wang F. (2024). Measurement of Cellular MDA Content through MTBE-Extraction Based TBA Assay by Eliminating Cellular Interferences. J. Pharm. Biomed. Anal..

[27] Zhao D., Yan M., Xu H., Liang H., Zhang J., Li M., Wang C. (2023). Antioxidant and Antiaging Activity of Fermented Coix Seed Polysaccharides on *Caenorhabditis elegans*. Nutrients.

[28] Chen J.-C., Wang R., Wei C.-C. (2023). Anti-Aging Effects of Dietary Phytochemicals: From *Caenorhabditis elegans*, *Drosophila melanogaster*, Rodents to Clinical Studies. Crit. Rev. Food Sci. Nutr..

[29] Lin Q., Song B., Zhong Y., Yin H., Li Z., Wang Z., Cheong K.-L., Huang R., Zhong S. (2023). Effect of *Sodium Hyaluronate* on Antioxidant and Anti-Ageing Activities in *Caenorhabditis elegans*. Foods.

[30] Zhang X., Liu L., Luo J., Peng X. (2022). Anti-Aging Potency Correlates with Metabolites from in Vitro Fermentation of Edible Fungal Polysaccharides Using Human Fecal Intestinal Microflora. Food Funct..

[31] Yen C.A., Curran S.P. (2016). Gene-Diet Interactions and Aging in *C. elegans*. Exp. Gerontol..

[32] Sharma G., Shin E.-J., Sharma N., Nah S.-Y., Mai H.N., Nguyen B.T., Jeong J.H., Lei X.G., Kim H.-C. (2021). Glutathione Peroxidase-1 and Neuromodulation: Novel Potentials of an Old Enzyme. Food Chem. Toxicol..

[33] Lee H.-B., Kim E.-K., Park S.-J., Bang S.-G., Kim T.G., Chung D.-W. (2010). Isolation and Characterization of Nicotiflorin Obtained by Enzymatic Hydrolysis of Two Precursors in Tea Seed Extract. J. Agric. Food Chem..

[34] Xu Y.-Q., Zhang Y.-N., Chen J.-X., Wang F., Du Q.-Z., Yin J.-F. (2018). Quantitative Analyses of the Bitterness and Astringency of Catechins from Green Tea. Food Chem..

[35] Huang Y., He M., Zhang J., Cheng S., Cheng X., Chen H., Wu G., Wang F., Zeng S. (2024). White Tea Aqueous Extract: A Potential Anti-Aging Agent Against High-Fat Diet-Induced Senescence in *Drosophila melanogaster*. Foods.

[36] Chen T., Luo S., Wang X., Zhou Y., Dai Y., Zhou L., Feng S., Yuan M., Ding C. (2021). Polyphenols from *Blumea laciniata* Extended the Lifespan and Enhanced Resistance to Stress in *Caenorhabditis elegans* via the Insulin Signaling Pathway. Antioxidants.

[37] Mokra D., Joskova M., Mokry J. (2022). Therapeutic Effects of Green Tea Polyphenol (‒)-Epigallocatechin-3-Gallate (EGCG) in Relation to Molecular Pathways Controlling Inflammation, Oxidative Stress, and Apoptosis. Int. J. Mol. Sci..

[38] Zhao T., Li C., Wang S., Song X. (2022). Green Tea (*Camellia sinensis*): A Review of Its Phytochemistry, Pharmacology, and Toxicology. Molecules.

[39] Zhao X., Song J.-L., Yi R., Li G., Sun P., Park K.-Y., Suo H. (2018). Comparison of Antioxidative Effects of Insect Tea and Its Raw Tea (Kuding Tea) Polyphenols in Kunming Mice. Molecules.

[40] Cheng S.-C., Li W.-H., Shi Y.-C., Yen P.-L., Lin H.-Y., Liao V.H.-C., Chang S.-T. (2014). Antioxidant Activity and Delayed Aging Effects of Hot Water Extract from *Chamaecyparis obtusa* var. formosana leaves. J. Agric. Food Chem..

[41] Tungmunnithum D., Drouet S., Hano C. (2022). Flavonoids from Sacred Lotus Stamen Extract Slows Chronological Aging in Yeast Model by Reducing Oxidative Stress and Maintaining Cellular Metabolism. Cells.

[42] Agraharam G., Girigoswami A., Girigoswami K. (2022). Myricetin: A Multifunctional Flavonol in Biomedicine. Curr. Pharmacol. Rep..

[43] Büchter C., Ackermann D., Honnen S., Arnold N., Havermann S., Koch K., Wätjen W. (2015). Methylated Derivatives of Myricetin Enhance Life Span in *Caenorhabditis elegans* Dependent on the Transcription Factor DAF-16. Food Funct..

[44] Cavalier A.N., Clayton Z.S., Wahl D., Hutton D.A., McEntee C.M., Seals D.R., LaRocca T.J. (2024). Protective Effects of Apigenin on the Brain Transcriptome with Aging. Mech. Ageing Dev..

[45] Clayton Z.S., Hutton D.A., Brunt V.E., VanDongen N.S., Ziemba B.P., Casso A.G., Greenberg N.T., Mercer A.N., Rossman M.J., Campisi J. (2021). Apigenin Restores Endothelial Function by Ameliorating Oxidative Stress, Reverses Aortic Stiffening, and Mitigates Vascular Inflammation with Aging. Am. J. Physiol. Heart Circ. Physiol..

[46] Kramer D.J., Johnson A.A. (2024). Apigenin: A Natural Molecule at the Intersection of Sleep and Aging. Front. Nutr..

[47] Zumerle S., Sarill M., Saponaro M., Colucci M., Contu L., Lazzarini E., Sartori R., Pezzini C., Rinaldi A., Scanu A. (2024). Targeting Senescence Induced by Age or Chemotherapy with a Polyphenol-Rich Natural Extract Improves Longevity and Healthspan in Mice. Nat. Aging.

[48] Akter R., Afrose A., Rahman M.R., Chowdhury R., Nirzhor S.S.R., Khan R.I., Kabir M.T. (2021). A Comprehensive Analysis into the Therapeutic Application of Natural Products as SIRT6 Modulators in Alzheimer’s Disease, Aging, Cancer, Inflammation, and Diabetes. Int. J. Mol. Sci..

[49] Guo P., Wang P., Liu L., Wang P., Lin G., Qu Z., Yu Z., Liu N. (2023). Naringin Alleviates Glucose-Induced Aging by Reducing Fat Accumulation and Promoting Autophagy in *Caenorhabditis elegans*. Nutrients.

[50] Ge Y., Chen H., Wang J., Liu G., Cui S.W., Kang J., Jiang Y., Wang H. (2021). Naringenin Prolongs Lifespan and Delays Aging Mediated by IIS and MAPK in *Caenorhabditis elegans*. Food Funct..

[51] Wang R., Ding A., Wang J., Wang J., Zhou Y., Chen M., Ju S., Tan M., Xiang Z. (2024). Astragalin from Thesium Chinense: A Novel Anti-Aging and Antioxidant Agent Targeting IGFR/CD38/Sirtuins. Antioxidants.

[52] Büchter C., Ackermann D., Havermann S., Honnen S., Chovolou Y., Fritz G., Kampkötter A., Wätjen W. (2013). Myricetin-Mediated Lifespan Extension in *Caenorhabditis elegans* Is Modulated by DAF-16. Int. J. Mol. Sci..

[53] Tao M., Li R., Zhang Z., Wu T., Xu T., Zogona D., Huang Y., Pan S., Xu X. (2022). Vitexin and Isovitexin Act through Inhibition of Insulin Receptor to Promote Longevity and Fitness in *Caenorhabditis elegans*. Mol. Nutr. Food Res..

[54] Lashmanova E., Zemskaya N., Proshkina E., Kudryavtseva A., Volosnikova M., Marusich E., Leonov S., Zhavoronkov A., Moskalev A. (2017). The Evaluation of Geroprotective Effects of Selected Flavonoids in *Drosophila melanogaster* and *Caenorhabditis elegans*. Front. Pharmacol..

