# Supplementary figures and images for: Characterization and Anti-Aging Potency of Phenolic Compounds in Xianhu Tea Extracts
Source: Foods. 2025 Feb 21;14(5):737. doi: 10.3390/foods14050737 (PMC11899101; doi:10.3390/foods14050737)

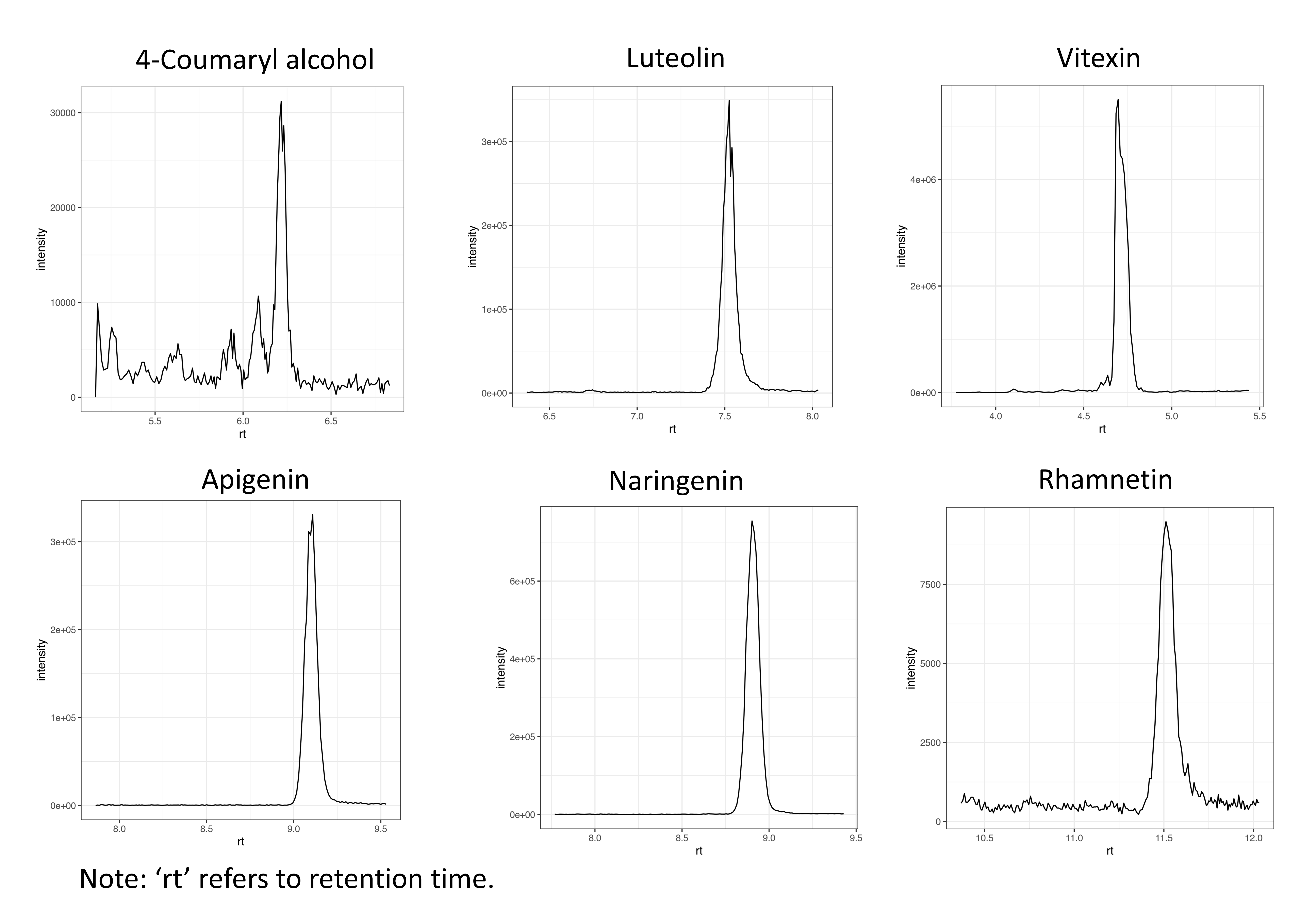

Supplement: Supplementary file 1 [file foods-14-00737-s001.zip › Figure S1.TIF]

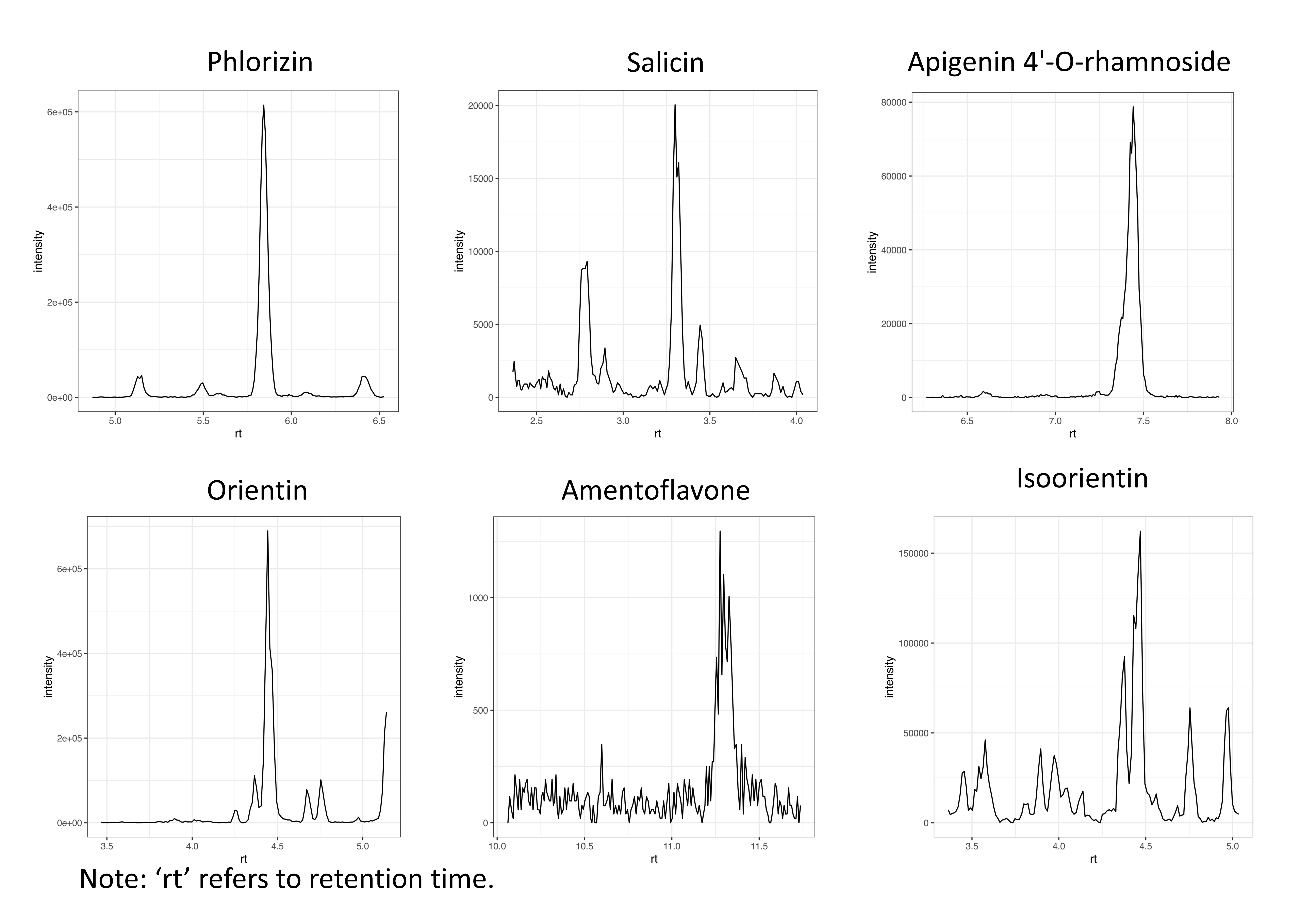

Supplement: Supplementary file 1 [file foods-14-00737-s001.zip › Figure S2.TIF]

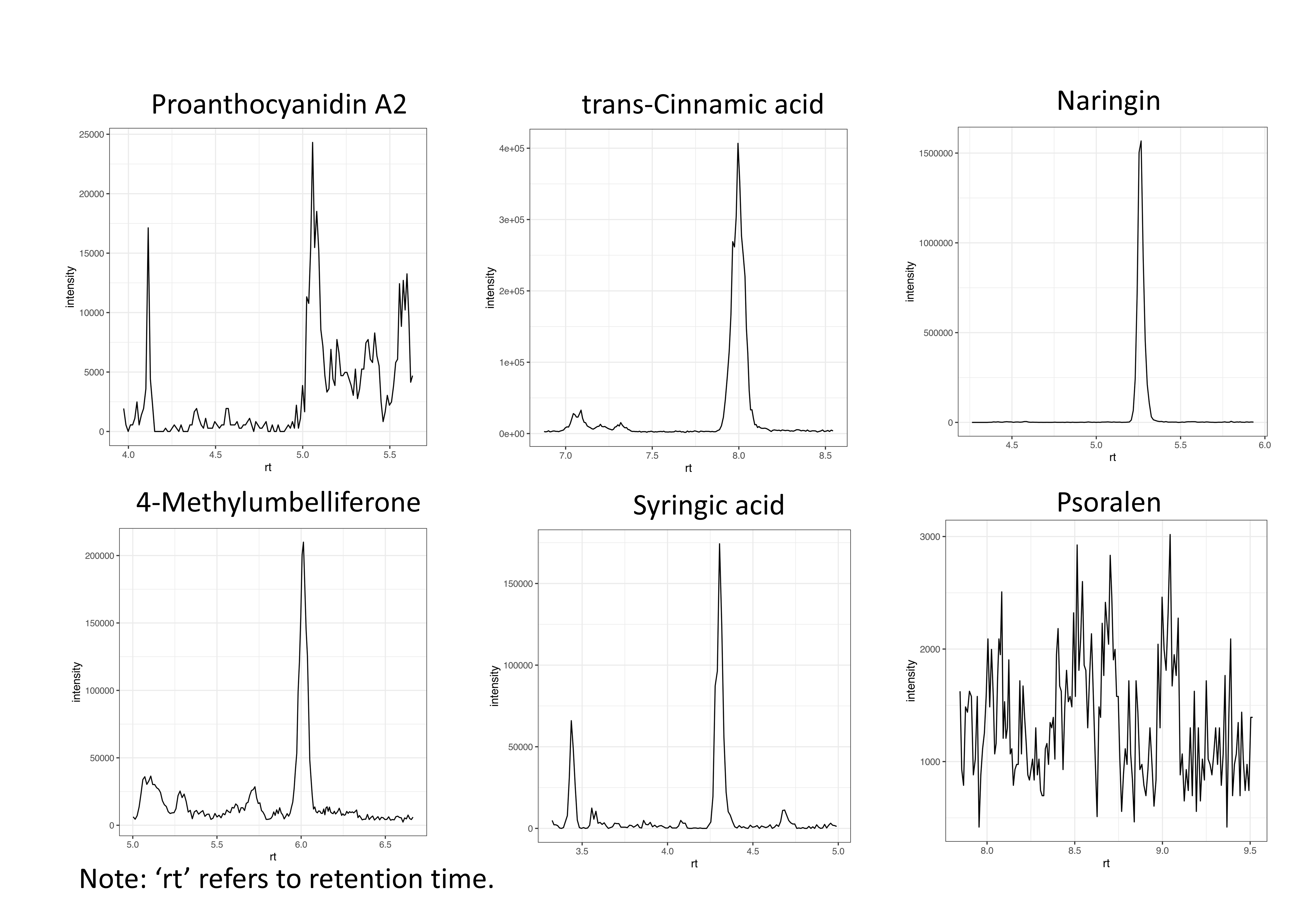

Supplement: Supplementary file 1 [file foods-14-00737-s001.zip › Figure S3.TIF]

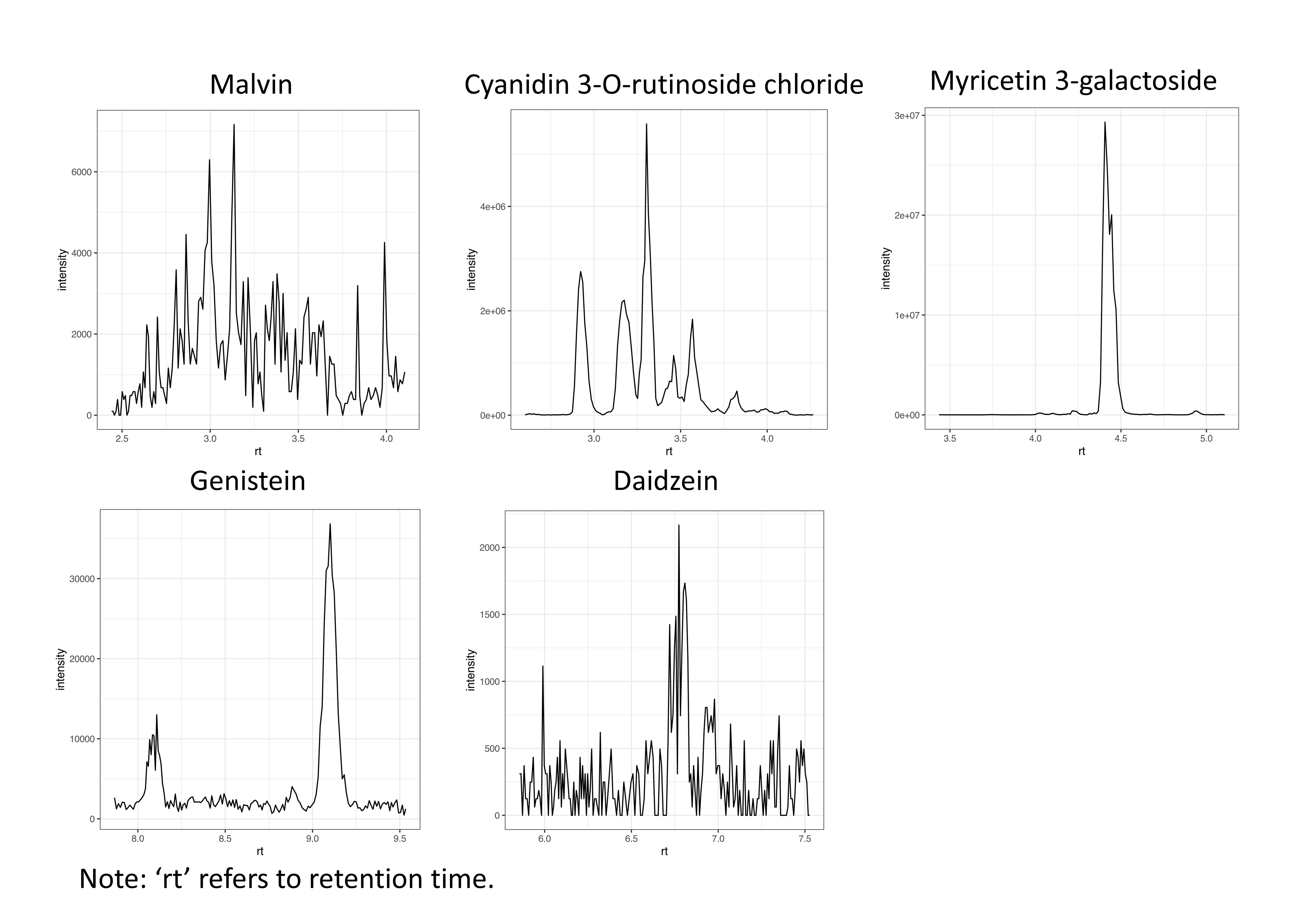

Supplement: Supplementary file 1 [file foods-14-00737-s001.zip › Figure S4.TIF]
